# Ant Larval Demand Reduces Aphid Colony Growth Rates in an Ant-Aphid Interactio

**DOI:** 10.3390/insects3010120

**Published:** 2012-02-02

**Authors:** Tom H. Oliver, Simon R. Leather, James M. Cook

**Affiliations:** 1 Division of Biology, Imperial College London, Silwood Park Campus, Ascot, Berkshire SL5 7PY, UK; 2 Centre for Ecology and Hydrology, Crowmarsh Gifford, Wallingford, Oxfordshire, OX10 8BB, UK; 3 School of Biological Sciences, University of Reading, Reading, RG6 6AS, UK; E-Mails: s.leather@imperial.ac.uk (S.R.L.); james.cook@reading.ac.uk (J.M.C.)

**Keywords:** context dependency, conditionality, mutualism, ant predation, keystone interaction, *Lasius niger*, *Aphis fabae*

## Abstract

Ants often form mutualistic interactions with aphids, soliciting honeydew in return for protective services. Under certain circumstances, however, ants will prey upon aphids. In addition, in the presence of ants aphids may increase the quantity or quality of honeydew produced, which is costly. Through these mechanisms, ant attendance can reduce aphid colony growth rates. However, it is unknown whether demand from within the ant colony can affect the ant-aphid interaction. In a factorial experiment, we tested whether the presence of larvae in *Lasius niger* ant colonies affected the growth rate of *Aphis fabae* colonies. Other explanatory variables tested were the origin of ant colonies (two separate colonies were used) and previous diet (sugar only or sugar and protein). We found that the presence of larvae in the ant colony significantly reduced the growth rate of aphid colonies. Previous diet and colony origin did not affect aphid colony growth rates. Our results suggest that ant colonies balance the flow of two separate resources from aphid colonies- renewable sugars or a protein-rich meal, depending on demand from ant larvae within the nest. Aphid payoffs from the ant-aphid interaction may change on a seasonal basis, as the demand from larvae within the ant colony waxes and wanes.

## 1. Introduction

Ants (Hymenoptera: Formicidae) often engage in mutualistic interactions with aphids (Hemiptera: Sternorrhyncha, previously Homoptera; [1]), receiving carbohydrate-rich honeydew in return for a number of protective and sanitary services [2,3,4]. In this symbiotic interaction, behavioural and physiological adaptations in both species groups ensure that interacting individuals are often co-located in space and time. For example, evolutionary adaptations in ants range from herding aphids using both physical means (e.g., pulling off alate aphid wings; Kunkel 1973, as cited by [5]) and chemical means (e.g., suppressing alate aphid development; [6]), and even transporting aphids to new feeding sites [7,8,9]. Evolutionary adaptations in aphids include a reduced movement response to aphid alarm pheromones when in the presence of ants [10], an ability to modify honeydew composition [11,12,13] and, possibly, reduced apterous dispersal behavior (although it is still unconfirmed whether this is adaptive for aphids or is an extended phenotype of ants, *i.e.*, through chemical manipulation; [14]).

The symbioses between ants and aphids, however, are not static but in a continual state of flux, depending on the changing distributions and densities of interacting species [15,16,17]. In certain circumstances, costs may exceed benefits causing the net effect of the interaction to switch sign from mutualism to antagonism, with potentially important consequences for community dynamics [18,19,20]. The key point is that each organism evolves to maximise its own benefit within the interaction. Physiological and behavioural innovations may arise which allow organisms to ‘fine tune’ interactions and derive maximal benefits. For example, aphids have evolved to modify the quality and quantity of honeydew in order to alter its attractiveness to ants [11,12,13]. Honeydew forms an important part of the diet for many ant species and its availability can structure ant communities [21,22,23,24,25]. In contrast, ants may maximise benefits by changing their behaviour towards aphids; for example, increasing predatory- rather than tending- behaviour.

Previous research shows that ants may switch from tending aphids to preying upon them if there are alternate sources of carbohydrate available to ants [26]. It has also been suggested that predation rates may increase if protein-rich prey sources are scarce [27], although a study by Offenberg [26] did not support this hypothesis. Offenberg found that alternative protein sources offered to ants did not affect aphid predation rates. Offenberg’s study did not consider the effect of protein supply together with demand of ant larvae in the colony, however. In colony developmental stages where ant larvae are present, protein may be more important for colony growth [28]. Colonies with larvae present often up-regulate foraging for protein food sources [29]. Hence, ants might increase predation of aphids unless alternative protein sources are provided.

In this study we investigated whether the presence of larvae in ant colonies, and previous diets fed to ants, affected aphid fitness. Our aim was to manipulate the developmental or physiological cues detected by ant workers. We measured the fitness effects on aphids in terms of colony reproductive success, a measure of Darwinian fitness [30].

Changes to parthenogenetic aphid colony growth rates may drive ecological and evolutionary changes in aphid-ant interactions by modifying selection pressure for mutualistic or antagonistic traits [31,32]. Our hypothesis was that the nutritional status of ant colonies can affect their behaviour towards—and the subsequent fitness of—tended aphid colonies [2]. Specifically, we test whether increasing ant colony protein demand through the presence of larvae will have a deleterious effect on aphid colony growth rates and hence switch the interaction from mutualism or commensalism towards parasitism.

## 2. Materials and Methods

We assessed the effects of ants on the fitness of mutualistic aphids using ants fed on different diets and kept with larvae present or absent. Two *Lasius niger* L. colonies, with brood and queens, were excavated from the grounds of Silwood Park, Ascot, UK in October 2006, and each stored in an individual five-litre plastic container (450–750 ants in each) with soil, moist cotton wool and a test tube containing sucrose solution with a cotton wool bung. *Lasius niger* is a common ant in temperate ecosystems, especially on disturbed ground and gardens. Foraging ants are recruited to food sources via pheromone trails left by scouts [33,34]. The species feeds on insect prey but also solicit honeydew from Sternorrhyncha [2,35], which may provide much of the ants’ nutritional requirements in terms of proteins (from amino acids) and carbohydrates [2]. The interaction with *Aphis fabae* Scopoli is generally considered to be mutualistic although predation by ants often occurs, and laboratory experiments often reveal a net parasitic effect on aphids, probably due to the lack of natural enemies in experimental set-ups [36,37,38,39]. Colonies were overwintered by storing in a refrigerated room at 5 °C. Before bringing back to room temperature in February or April, colonies were kept at 10 °C for one week [40]. Ants were then placed into forty eight 20 × 10 cm plastic containers with 20 workers per box. Each box contained an upturned plastic plant saucer for ants to gather beneath, a test tube with sucrose solution and two small open plastic boxes containing water and moist cotton wool.

**Figure 1:**
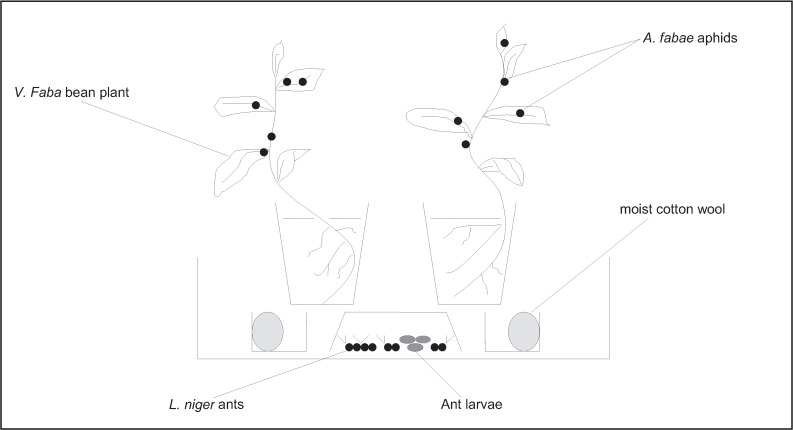
Experimental set up. Ants are free to collect honeydew or prey upon aphids from the plants.

During the experiment, the 20 ants in each box were allowed access to two *Vicia faba* L. bean plants, upon which five fourth instar *A. fabae* aphids were placed at the start of the experiment (Figure 1). The aphids were collected from Silwood Park UK and kept in culture for two years on *V. faba* plants in netted cages. The plants used in experiment were selected seedlings all ca. 10 cm tall (*i.e.*, about 20 days old) and grown with the same watering regime in standard potting compost in the greenhouse. Plants were from different parents and, were therefore, randomly distributed among the three treatments because plant genotype can affect ant-aphid interactions [19]. A week before the experiment the plants were removed from soil and the roots washed. For a separate study considering host plant quality (unpublished data), one plant was placed in de-ionised water and one in rainwater. However, the current study focuses only on the effects of different ant treatments, rather than differences between plants, therefore, when referring to aphid counts we henceforth refer to the sum total of aphids on both plants. For each box, the number of foraging ants on plants and the total number of aphids surviving on both plants were recorded at midday on seven consecutive days.

Ant treatments were as follows: Colony origin (two different colonies), Larval presence (20 larvae present, or larvae absent), Diet (protein and sugar, or sugar only). To mimic different developmental stages, the experimental colonies, which were all queenless, were either kept with larvae during the winter and throughout the experiment, or larvae were absent with workers only present. At the start of the experiment, larvae were in the intermediate stage of development, approximately 2.5–3.5 mm long. For the different dietary regimes, some ants were fed sugar (20% sucrose ad libitum) and twice a week protein in the form of chopped *Calliphora* larvae (Diptera: Calliphoridae) (Crowthorne Fishing Supplies, Berkshire, UK), while the remaining ants subsisted on sugar alone. Each combination of factor levels was tested, which amounted to eight treatment combinations, and with six replicates of each, to 48 boxes in total. In addition, 36 replicates of a control treatment, with aphids and plants but no ants present, were conducted.

**Figure 2:**
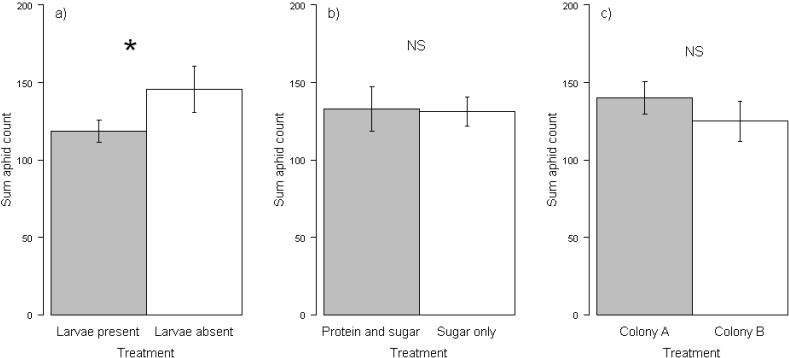
Mean total number of aphids surviving on plants under different treatments: (**a**) with 20 ant larvae present or absent in the colony, (**b**)ants fed on protein and sugar or sugar only, or (**c**) different colony origin. Bars represent standard errors of the means. Variance around means was high as a consequence of the factorial experimental design (see methods). Only larval presence had a significant effect on aphid fitness, indicated by an asterisk.

Due to space and time constraints, only 14 boxes were tested per week over six weeks. To achieve a fair test, these 14 boxes comprised the complete combination of factor levels (eight boxes) plus six control boxes, and time was included as a blocking factor in the analysis. For the first three experimental ‘blocks’, ant treatments were initially set up on diets in February and testing with aphids began in March. Thus, ants were kept on diet for four, five or six weeks before experimentation. For the final three experimental ‘blocks’, ant treatments were removed from refrigeration and set up in March. Tests began again four, five or six weeks later. Ant survival was assessed at the end of the experiment. Zero to five individuals died in each colony; however these deaths were random with respect to the treatments tested.

### 2.1. Statistical Analysis

We analysed whether ant treatments (diet, larvae and colony origin) differentially affected overall aphid reproductive success per box. Aphid reproductive success per box was measured as the sum total aphid count on both host plants after seven days. To test for the overall effect of ants we initially compared the sum aphid count in control boxes with boxes containing ants using a Students t-test with a Welch approximation for degrees of freedom due to unequal variances (boxes with ants had greater variance than controls). We next investigated whether the different ant treatments differentially affected aphid reproductive success. To do this, the control boxes were excluded and the ant treatments: colony origin, larval presence and diet, and all interaction effects, were included as fixed effects in a mixed effects model. The week (one to six) that the experiment was conducted was included as a random effect. We also analysed whether the ant treatments led to differences in the number of ants foraging on plants. To analyse ant counts on plants, the response variable was the mean number of ants foraging on plants per box over the seven days.

Data were analysed using linear mixed effects models in the program R [41] using the *lme4* package [42]. The response variable was natural log transformed to achieve normality (tested using a Shapiro-Wilk normality test). Significance values of model coefficients were estimated using Markov Chain Monte Carlo simulations with 10^4^ iterations. During the experiment, a few plants died for no apparent reason, severely reducing aphid counts. If this occurred, data from the entire box were excluded from analysis. Thus, in total six boxes were excluded, resulting in a final total sample size of 42 ant treatment boxes with 36 control boxes.

## 3. Results and Discussion

### 3.1. Results

The effect of three ant treatments, colony origin, larval presence and diet, on the reproductive success of ant-tended aphids was investigated. *Lasius niger* ants were observed to forage on the plants and tended aphids by collecting honeydew and carrying it to the nest. After seven days, there was no significant difference in the total number of aphids on ant treatment plants *versus* control treatment plants (sum aphids in ant treatment boxes: 132.07 ± 8.47 [SE] aphids; control: 147.41 ± 6.86 [SE] aphids; t = 1.41, df = 74.51, p = 0.163). There were significant differences between the ant treatments on aphid fitness, however. Ant colonies with larvae present were associated with significantly fewer aphids than ant colonies without larvae present (Figure 2; Table S1). Indeed the difference in total aphid counts between ant treatments and the control were driven primarily by ant colonies with larvae present; ant colonies with larvae present had significantly fewer aphids than the control treatment (ant treatment with larvae: 118.52 ± 7.37 [SE] aphids, control: 147.41 ± 6.86 [SE] aphids, t = 2.87, df = 48.73, p = 0.006), whilst ant colonies without larvae present were not significantly different from the control (ant treatment without larvae: 145.6 ± 14.88 [SE] aphids, control: 147.41 ± 6.86 [SE] aphids, t = 0.11, df = 28.67, p = 0.913). Thus, larval presence was a significant predictor of aphid fitness. Ant colony origin did not have a significant effect on aphid fitness, nor did the previous diet fed to ants (protein and sugar or sugar only) (Table S1). There were no significant interaction effects between any of the explanatory variables.

The mean number of ants on plants over the seven day recording period was not affected by any of the experimental treatments. The largest difference in ant foragers was between different ant colonies; however these differences were not significant (Table S2).

### 3.2. Discussion

In this study we investigated how the presence of larvae in ant colonies, and the previous diets fed to ants, affected aphid colony growth rates in an ant-aphid interaction. We found that the presence of larvae in the ant colony significantly reduced aphid colony growth rates. In contrast, the previous diet fed to ants and the origin of ant colonies did not significantly alter aphid colony growth rates.

A study by Offenberg [26] showed that although the presence of alternative sugar sources in the environment increased the predation of aphids by ants, alternative protein sources had little effect on ant behaviour. Our study supports this finding, and we also show that the presence of ant larvae within the colony has no effect on this result, *i.e.*, there was no significant interaction between the presence of larvae and previous diet on aphid colony growth rates.

There was, however, a strong main effect of ant larvae on the growth of aphid colonies. The presence of larvae in ant colonies significantly reduced the growth rate of aphid colonies. There may be a number of different reasons for aphid colony reproductive success to change in response to the presence of ant larvae within the colony. Firstly, ants might change their tending behaviour leading to increased dispersal of aphids from plants. We think this explanation unlikely as there were similar numbers of ants tending aphids irrespective of larval presence (Table S2). Ants are well adapted to maintain aphid colonies together, often using chemicals to reduce aphid dispersal away from the colony [6,14]. Our second explanation is that ant workers from colonies containing brood may solicit increased volumes, or better quality, honeydew from aphids [12,13,35,43,44,45], therefore aphids are forced to switch resources from reproduction to honeydew production. Presumably, aphids might make this switch because of the threat of predation by ants. Aphids producing honeydew are chemically marked by ants, and aphids without chemical markings are more likely to be preyed upon [46]. Finally, ants might reduce aphid colony fitness through direct predation [26,47]. Larval development is known to place a greater requirement on the colony for protein-based food [28,29], which ants might satisfy by eating rather than tending aphids. Predation of aphids was not directly observed in this experiment, but *L. niger* is well known to occasionally use *A. fabae* as prey ([38]; T.H. Oliver personal observation).

In this experiment, we did not find the expected interaction effect whereby aphid colony growth decreased when tended by ant colonies with larvae *and* when extra protein was not supplied to ants. One reason for this may be that the protein was not in a suitable form or type that the ants required. However, the lack of a protein effect was also found by Offenberg [26] with *Lasius niger* ants fed with freeze-killed *Drosophila melanogaster*, *Musca domestica* L. or *Tenebrio molitor* L.. Therefore, rather than ants with larvae present needing more protein and increasing predation upon aphids, perhaps our former explanation is more likely: that ant colonies with larvae have increased demand for carbohydrates. This may cause them to modify their behaviour towards aphids and subsequently cause the aphids to increase honeydew production at the cost of reduced colony growth.

For temperate ants, the number of larvae in an ant colony changes on a seasonal basis [5]. At the start of the year workers awaken from overwinter dormancy and begin to forage for food. It is likely that sugar is very valuable to the colony at this point, as it is used as a fuel to feed workers which further increases foraging efficiency [48,49]. As larvae develop from eggs, however, there is likely to be a growing demand for food. It is not clear that protein demand is the primary driver affecting ant behaviour towards tended aphids when ant larvae are present in the colony; however, the presence of ant larvae does lead to consequent decreases in aphid colony reproductive rates. Further research is needed to address whether this is through increased predation of aphids or through increased honeydew production by aphids. If the results from our experiment transfer to the field, then the behaviour of ants towards aphids will change as the demand from larvae in the colony increases. Through the threat of predation, or direct predation itself, ants may extract more resources from aphids, with consequent suppression of aphid colony growth rates during these periods. Hence, our study demonstrates how colony state can affect the behaviour of interacting species and cause temporal variation in costs and benefits and even the quantitative outcome of species interactions (e.g., potentially shifting interactions from mutualism to commensalism or parasitism; [15,16,17]).

## 4. Conclusions

To summarise, our results show that larval presence in ant colonies reduces the growth rates of aphid colonies in an ant-aphid interaction. The mechanism may be direct predation of aphids by ants, or communication of a threat of increased predation leading to increased production of aphid honeydew. Whatever mechanism dominates, it appears that ants balance both the supply of carbohydrates in the environment and demand for nutrients from within the colony to determine their behaviour towards aphids. Aphids may be tended and protected as renewable (mainly sugar) resources or used as a protein-rich meal. From the results of this study we predict that aphid rewards from the ant-aphid interaction will change on a seasonal basis, as the demand from larvae within the ant colony waxes and wanes.

## Supplementary Material

**Table S1:** Effects of different ant treatments on the total numbers of aphids in boxes after seven days. Significant p-values (*p* < 0.05) using Markov chain Monte-Carlo simulations are highlighted in bold.

Explanatory variable	*n*	Mean aphids per box	SE	*t*	p
**Diet**				0.19	0.87
Sugar and protein	21	132.95	14.28		
Sugar only	21	131.19	9.48		
**Larval presence**				2.48	**0.029**
Larvae present	21	118.52	7.37		
Larvae absent	21	145.62	14.88		
**Colony**				1.93	0.073
one	20	139.85	10.48		
two	22	125.00	13.10		
**Control**	36	147.42	6.86	-	-

**Table S2:** Effects of different ant treatments on the mean number of ants foraging on plants over seven days. There were no significant differences between treatments.

Explanatory variable	*n*	Mean ants per box	SE	*t*	p
**Diet**				0.41	0.72
Sugar and protein	21	0.32	0.16		
Sugar only	21	0.43	0.22		
**Larval presence**				0.17	0.83
Larvae present	21	0.33	0.15		
Larvae absent	21	0.41			
**Colony**				0.94	0.35
one	20	0.23	0.16		
two	22	0.50	0.22		

## References

[1] Carver M., Gross G.F., Woodward T.E., Naumann I.D., Carne P.B., Lawrence J.F., Nielsen E.S., Spradberry P., Taylor R.W., Whitten M.J., Littlejohn M.J. (1991). Hemiptera. The Insects of Australia.

[2] Way M.J. (1963). Mutualism between ants and honeydew producing Homoptera. Annu. Rev. Entomol..

[3] Stadler B., Dixon A.F.G. (2005). Ecology and evolution of aphid- ant interactions. Annu. Rev. Ecol. Syst..

[4] Styrsky J.D., Eubanks M.D. (2007). Ecological consequences of interactions between ants and honeydew producing insects. Proc. R. Soc. B.

[5] Hölldobler B., Wilson E.O. (1990). The Ants.

[6] Kleinjan J.E., Mittler T.E. (1975). A chemical influence of ants in wing development in aphids. Entomol. Exp. Appl..

[7] Jones C.R. (1929). Ants and their relation to aphids. Bulletin (Colarado Agricultural Experiment Station).

[8] Sudd J.H. (1967). An Introduction to the Behaviour of Ants.

[9] Collins C.M., Leather S.R. (2002). Ant-mediated dispersal of the black willow aphid *Pterocomma salicis* L.; Does the ant *Lasius niger* L. judge aphid-host quality. Ecol. Entomol..

[10] Nault L.R., Montgomery M.E., Bowers W.S. (1976). Ant-aphid association: Role of aphid alarm pheromone. Science.

[11] Fischer M.K., Shingleton A.W. (2001). Host plant and ants influence the honeydew sugar composition of aphids. Funct. Ecol..

[12] Yao I., Akimoto S. (2001). Ant attendance changes the sugar composition of the honeydew of the drepanosiphid aphid *Tuberculatus quercicola*. Oecologia.

[13] Yao I., Akimoto S. (2002). Flexibility in the composition and concentration of amino acids in honeydew of the drepanosiphid aphid *Tuberculatus quercicola*. Ecol. Entomol..

[14] Oliver T.H., Mashanova A., Leather S.R., Cook J.M., Jansen V.A.A. (2007). Ant semiochemicals limit apterous aphid dispersal. Proc. R. Soc. B.

[15] Cushman J.H., Whitham T.G. (1989). Conditional mutualism in a membracid-ant association: Temporal, age-specific and density dependent effects. Ecology.

[16] Cushman J.H. (1991). Host-plant mediation of insect mutualisms: Variable outcomes in herbivore-ant interactions. Oikos.

[17] Bronstein J.L. (1994). Conditional outcomes in mutualistic interactions. Trends Ecol. Evol..

[18] Muller C.B., Godfray C. (1999). Predators and mutualists influence the exclusion of aphid species from natural communities. Oecologia.

[19] Wimp G.M., Whitham T.G. (2001). Biodiversity consequences of predation and host plant hybridization on an aphid- ant mutualism. Ecology.

[20] Savage A.M., Peterson M.A. (2007). Mutualism in a community context: The positive feedback between an ant-aphid mutualism and a gall-making midge. Oecologia.

[21] Lach L., Hobbs E.R., Majer E.J.D. (2009). Herbivory-induced extraﬂoral nectar increases native and invasive ant worker survival. Popul. Ecol..

[22] Blüthgen N., Verhaagh M., Goitia W., Jaffé K., Morawetz W., Barthlott W. (2000). How plants shape the ant community in the Amazonian rainforest canopy: The key role of extrafloral nectaries and homopteran honeydew. Oecologia.

[23] Oliver T.H., Leather S.R., Cook J.M. (2008). Macroevolutionary patterns in the origin of mutualisms involving ants. J. Evol. Biol..

[24] Oliver T.H., Pettitt T., Leather S.R., Cook J.M. (2008). Numerical abundance of invasive ants and monopolisation of exudate-producing resources- a chicken and egg situation. J. Insect Conserv. Divers..

[25] Byk J., Del-Claro K. (2010). Ant-plant interaction in the neotropical savanna: Direct beneﬁcial effects of extraﬂoral nectar on ant colony ﬁtness. Popul. Ecol..

[26] Offenberg J.H. (2001). Balancing between mutualism and exploitation: The symbiotic interaction between *Lasius* ants and aphids. Behav. Ecol. Sociobiol..

[27] Pontin A.J. (1958). A preliminary note on the eating of aphids by ants of the genus *Lasius*. Entomol. Month. Mag. (Lond.).

[28] Carroll C.R., Janzen D.H. (1973). The ecology of foraging ants. Annu. Rev. Ecol. Syst..

[29] Dussutour A., Simpson S.J. (2009). Communal nutrition in ants. Curr. Biol..

[30] Deutsch C.A., Tewksbury J.J., Huey R.B., Sheldon K.S., Ghalambor C.K., Haak D.C., Martin P.R. (2008). Impacts of climate warming on terrestrial ectotherms across latitude. Proc. Natl. Acad. Sci. USA.

[31] Dixon A.F.G. (1998). Aphid Ecology: An Optimisation Approach.

[32] Oliver T.H., Leather S.R., Cook J.M. (2009). Tolerance traits and the stability of mutualism. Oikos.

[33] Beckers R., Deneuborg J.L., Goss S. (1992). Trail laying behaviour during food recruitment in the ant Lasius niger (L.). Insectes Sociaux.

[34] Portha S., Deneuborg J.-L., Detrain C. (2004). How food type and brood influence foraging decisions of Lasius niger scouts. Anim. Behav..

[35] Fischer M.K., Volkl W., Hoffman K.H. (2005). Honeydew production and honeydew sugar composition of polyphagous black bean aphid, *Aphis fabae* (Hemiptera: Aphididae) on various host plants and implications for ant-attendance. Eur. J. Entomol..

[36] Banks C.J. (1958). Effects of the ant, *Lasius niger* L., on the behaviour and reproduction of the black bean aphid, *Aphis fabae* Scop. Bull. Entomol. Res..

[37] Banks C.J., Nixon H.L. (1958). Effects of the ant, *Lasius niger* L., on the feeding and excretion of the bean aphid, *Aphis fabae* Scop. J. Exp. Biol..

[38] Banks C.J. (1962). Effects of the ant *Lasius niger* L., on insects preying on small populations of *Aphis fabae* Scop. on bean plants. Ann. Appl. Biol..

[39] Oliver T.H., Cook J.M., Leather S.R. (2007). When are ant-attractant devices a worthwhile investment? *Vicia faba* extrafloral nectaries and *Lasius niger* ants. Popul. Ecol..

[40] Wardlaw J.C., Elmes G.W., Thomas J.A. (1998). Techniques for studying Maculinea butterflies: I. Rearing Maculinea caterpillars with Myrmica ants in the laboratory. J. Insect Conser..

[41] R Development Core Team (2007). R: A Language and Environment for Statistical Computing.

[42] Bates D., Maechler M., Dai B. (2008). lme4: Linear Mixed-Effects Models Using S4 Classes. R Package Version 0.999375–20.

[43] Takeda S., Kinomura K., Sakurai H. (1982). Effects of ant- attendance on the honeydew excretion and larviposition of the cowpea aphid, *Aphis craccivora*. Appl. Entomol. Zool..

[44] Del-Claro K., Oliveira P.S. (1993). Ant-Homoptera interactions: Do alternative sugar sources distract tending ants?. Oikos.

[45] Katayama N., Suzuki K. (2002). Cost and benefit of ant attendance for Aphis craccivora (Hemiptera: Aphididae) with reference to aphid colony size. Can. Entomol..

[46] Sakata H. (1994). How an ant decides to prey on or to attend aphids. Res. Popul. Ecol..

[47] Edinger B.B. (1985). Conditional mutualism in three aphid-tending ants. Bull. Ecol. Soc. Am..

[48] Sudd J.H., Sudd M.E. (1985). Seasonal changes in the response of wood-ants (*Formica lugubris*) to sucrose baits. Ecol. Entomol..

[49] Stadler B., Dixon A.F.G. (2008). Mutualism: Ants and Their Insect Partners.

